# Giant testicular germ‐cell tumours—An analysis of relative incidence and clinical features based on a clinical case series and a survey of the literature

**DOI:** 10.1002/bco2.70118

**Published:** 2026-01-12

**Authors:** Markus Angerer, Alexander C. Harms, Christian Wülfing, Klaus‐Peter Dieckmann

**Affiliations:** ^1^ Department of Urology Asklepios Klinik Altona Hamburg Germany; ^2^ Medizinisches Versorgungszentrum Hanse Histologikum Hamburg Germany

**Keywords:** diagnostic delay, giant tumour, nonseminoma, seminoma, testicular germ cell tumour, tumour size

## Abstract

**Objectives:**

This work aimed to document four new cases with giant testicular germ cell tumour (GCT) and to evaluate their relative incidence and clinical characteristics based on a literature survey. Despite the well‐established trend over time towards declining tumour sizes in testicular GCTs, giant testicular tumours (>15 cm in diameter) are still reported in present times.

**Patients, Methods:**

GCT patients treated during 2010–2025 were retrospectively evaluated with tabulating the following data: size of primary tumour (mm), age (years), histology, side and clinical stage (CS). The following parameters were calculated: relative frequency of giant GCTs; median tumour size in all GCTs and in various subgroups. A literature survey was conducted to identify previously published giant testicular GCTs followed by a descriptive evaluation of those cases.

**Results:**

Four (0.5%) giant GCTs were identified among 860 GCT patients, two seminomas and two nonseminomas, all having CS3 disease, two of whom were cured. The median tumour size was 32 mm in all GCTs, and 30 mm and 35 mm in seminomas (*n* = 541) and nonseminomas (*n* = 319), respectively. Median tumour size was significantly smaller in CS1 cases than in those with CS > 1 (32 mm vs. 38 mm). Of the 40 cases identified with the literature survey, 24 were nonseminomas, 62% were left‐sided, median age was 36 years, and 80% were cured. Diagnostic delay is the most frequent cause of excessive tumour growth.

**Conclusions:**

Giant testicular tumours are observed in 0.5% of all GCT patients while the median tumour size of 32 mm observed herein is consistent with current reports. In most cases of giant GCT, personal misapprehension of the swelling, lack of knowledge or shame appears to be the key element causing diagnostic delay and consequently, extraordinary tumour growth. Information campaigns including individuals from socioeconomically underprivileged groups could help to increase men's awareness of genital diseases.

## 1 INTRODUCTION

Reports of testicular growths from over a century ago often described exceptionally large neoplasms, aligning with the modern understanding of ‘giant tumours’. Nowadays, testicular germ cell tumours (GCTs) are the most common tumours in adolescents and young adults in western countries, encompassing 25% of all malignancies diagnosed in the age group of 20–40 years.^1^ In 1887, Kocher documented two testicular neoplasms measuring 23 and 35 cm in diameter, and another weighing 3.2 kg.^2^ Similarly, Monod and Terrillon's 1889 book on testicular diseases featured a hand‐painted image of an enormous tumour extending from the groin to mid‐thigh, though it lacked scale.^3^ Dew also illustrated several massive testicular tumours in 1925.^4^

However, historical accounts lacked an understanding of tumour biology, leaving it uncertain whether these cases involved GCTs or other neoplasms such as intrascrotal sarcomas, sex cord gonadal stromal tumours or spermatocytic tumours all of which can present with huge neoplastic masses.^4^, ^5^, ^6^

Our current understanding of the pathogenesis of testicular cancer largely stems from military doctors who treated numerous young soldiers with testis cancer during and after World War II.^7^, ^8^ Their observations established that the majority of testicular neoplasms originate from germ cells and that two subgroups of GCTs, seminomas and nonseminomas, are to be considered clinically.^9^, ^10^, ^11^

The first systematic assessment of primary tumour sizes was conducted by the British Testicular Tumour Panel in 1964. Among 400 seminoma cases, Thackray reported that 11% exceeded 10 times the normal testis size with the largest measuring 30 cm in diameter.^12^ Among 327 nonseminomas, Pugh noted 27% to be larger than four times the normal size of a testicle, with the largest reaching 16 cm in diameter.^13^

In the post‐world war decades, median tumour sizes of 5 and 5.7 cm, respectively, were reported from Western countries.^14^, ^15^ Since then, a continuous decrease in the median primary tumour sizes in both histologic subgroups has been observed worldwide with recent reports documenting median GCT sizes of 3, 3.5, 4.0, and 4.1 cm.^15^, ^16^, ^17^, ^18^, ^19^, ^20^, ^21^, ^22^, ^23^, ^24^

The decline in tumour size over recent decades remained largely unexplored, but several factors are likely to contribute. Reduced diagnostic delays in GCT patients were observed in multiple studies, suggesting that increased awareness among young adults has led to earlier detection.^19^, ^25^, ^26^, ^27^, ^28^ Additionally, advancements in diagnostic imaging, particularly ultrasound, have improved early identification, with up to 13% of testicular neoplasms now detected at sizes under 1 cm.^29^, ^30^, ^31^, ^32^ Despite public health efforts to raise awareness, reports of giant testicular tumours continue to emerge, even in recent years.^33^, ^34^, ^35^, ^36^, ^37^ These cases are typically isolated reports emphasizing their rarity and peculiarity. However, a comprehensive analysis of their frequency and clinical characteristics remains absent.

Therefore, the aim of the present work is to report four new cases with giant testicular GCTs and to assess their relative frequency in a large contemporary patient series. Also, to better understand the clinical characteristics of extraordinarily large testicular tumours, a comprehensive literature survey of such cases is provided.

## 2 PATIENTS, METHODS

### 2.1 Definition of giant testicular tumour

Despite numerous reports in the literature on giant testicular tumours, the condition remains poorly defined. Historically, the British Testicular Tumour Panel characterized tumour size relative to the normal testis.^13^ Some authors subsequently defined giant testicular tumours as those exceeding 10 times the volume of a normal testis.^38^, ^39^ However, this definition lacks precision and is subject to interpretation, leading to its limited adoption in subsequent investigations. To establish a clear and objective criterion, we define a giant testicular tumour as one with a diameter of ≥15 cm on gross pathological examination in an orchiectomy specimen. While this threshold is somewhat arbitrary, it probably serves as a practical classification, given the rarity of tumours exceeding this size. Moreover, from a clinical perspective, neoplasms considerably exceeding the size of 15 cm do not exhibit distinct pathological or prognostic features that would necessitate an additional category beyond ‘giant tumours’ (>15 cm).

### 2.2 Patients

We retrospectively evaluated consecutive patients treated for testicular GCTs in Albertinen Krankenhaus Hamburg, 2010–2017, and in Asklepios Klinik Altona, 2010–2025. Both institutions represent primary testicular cancer units serving the Greater Hamburg region offering all kinds of treatment except for high‐dose chemotherapy. The following data were derived from electronic hospital archives: patients' age (years), histology of primary tumour (seminoma [SE] or nonseminoma [NS]), laterality (left/right), clinical stage (CS) according to the EAU guide line^39^ and largest diameter of the tumour as stated in the gross pathological examination report. We excluded patients undergoing orchiectomy after upfront chemotherapy, those with primary extragonadal GCTs, burned‐out tumours and those with only germ cell neoplasia in situ. Bilateral disease was rated as two separate cases, if both neoplasms occurred within the study period. In patients undergoing orchiectomy in other institutions before receiving treatment in the two participating departments, the tumour sizes were derived from the original pathology reports. Around half of the patients had been included in other evaluations published previously.^21^, ^32^ In patients fulfilling the definition of giant tumours, further clinical details were secured, and the histopathological sections were re‐examined.

Ethical approval was provided by Ethikkommission der Ärztekammer Hamburg (PV7288, from 2 March 2020). Informed consent was obtained from patients or their parents in cases where images are included in this report. All actions of the present investigation accorded with the Declaration of Helsinki of the World Medical Association as amended by the 64th General Assembly, October 2013.

### 2.3 Statistical analysis

The clinical data were tabulated using a commercially available data base (MS Excel, version 2019). The relative frequency (%) with corresponding 95% confidence intervals (CIs) of giant tumours among the entire GCT population was calculated. The median sizes and interquartile ranges (IQRs) of tumour size were calculated for the entire GCT patient population and for the two histologic subgroups, SE and NS. Also, the relative frequencies of the following tumour‐size categories were calculated: <2 cm; 2.1–5 cm; 5.1–10 cm; 10.1–15 cm; >15 cm. Comparisons of median tumour sizes between subgroups were performed with the Mann–Whitney *U* test. Comparisons of frequencies were performed with the chi square test.

### 2.4 Literature survey

Electronic data bases PubMed and Google Scholar were searched for the term ‘giant testicular tumour’ or ‘giant testicular neoplasm’ supplemented by a hand search of reference lists of the identified papers. We included only reports published later than 1950, reporting GCT cases sized ≥15 cm or with descriptions compatible with this definition. We excluded giant tumours in undescended testis, giant intra‐abdominal GCTs and giant intrascrotal tumours of non‐germ‐cell origin. The cases identified were tabulated chronologically and then evaluated according to their clinical features.

## 3 RESULTS

### 3.1 Relative incidence and clinical features of giant tumours in the present cohort

**FIGURE 1:**
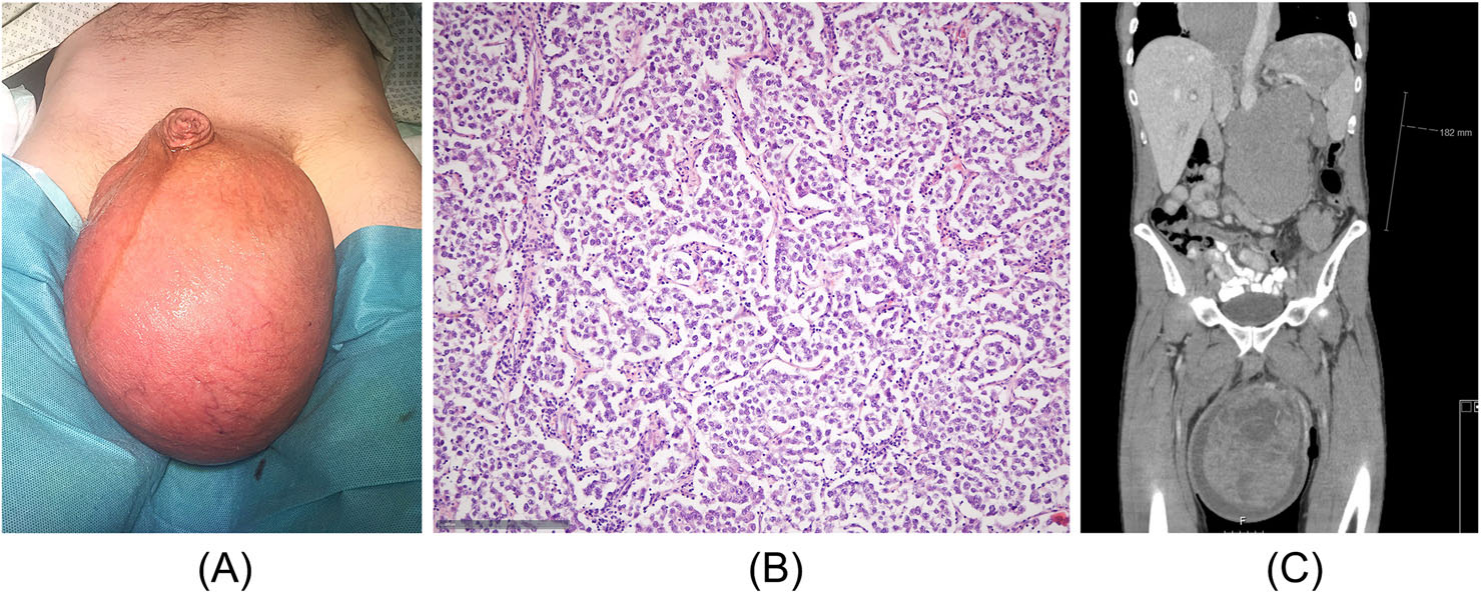
(A) Clinical appearance of left‐sided giant testicular tumour in a 52‐year‐old man (patient #1). (B) Histological finding of testicular specimen of the same patient, showing sheets and lobules of tumour cells separated by fibrous septae. The tumour cells are pale due to glycogen accumulation. The cell membranes tend to be well defined with distinct cell boundaries. There are scattered lymphocytes. Final histological diagnosis: seminoma. H&E staining, scale bottom left. (C) Computed tomography of same patient before orchiectomy showing large tumour masses in the scrotum, retroperitoneum and mediastinum, consistent with clinical stage III.

**FIGURE 2:**
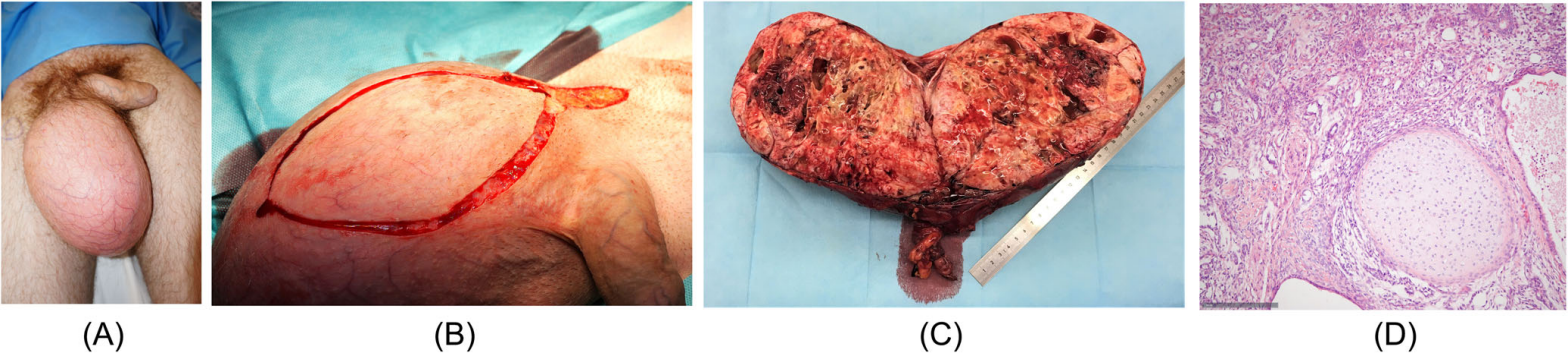
(A) Clinical appearance of right sided giant testicular tumour in a 20‐year‐old man (patient #2). (B) Surgical site during inguinal orchiectomy of same patient showing that the incision had to extended to the scrotum with excision of larger parts of the scrotal skin for tailoring superfluous skin. (C) Gross pathologic specimen of excised testis showing an inhomogeneous neoplastic mass with islands of cartilage, solid areas and cystic areas compatible with teratomatous elements in the tumour. (D) Histologic section of the orchiectomy specimen (same patient, #2) showing a lobule of mature cartilage and atypical gland‐like structures as well as pale tumour cells with small nuclei. Final histological diagnosis: mixed nonseminomatous tumour consisting of 80% teratoma and 20% yolk sac tumour. H&E staining, scale bottom left.

**FIGURE 3:**
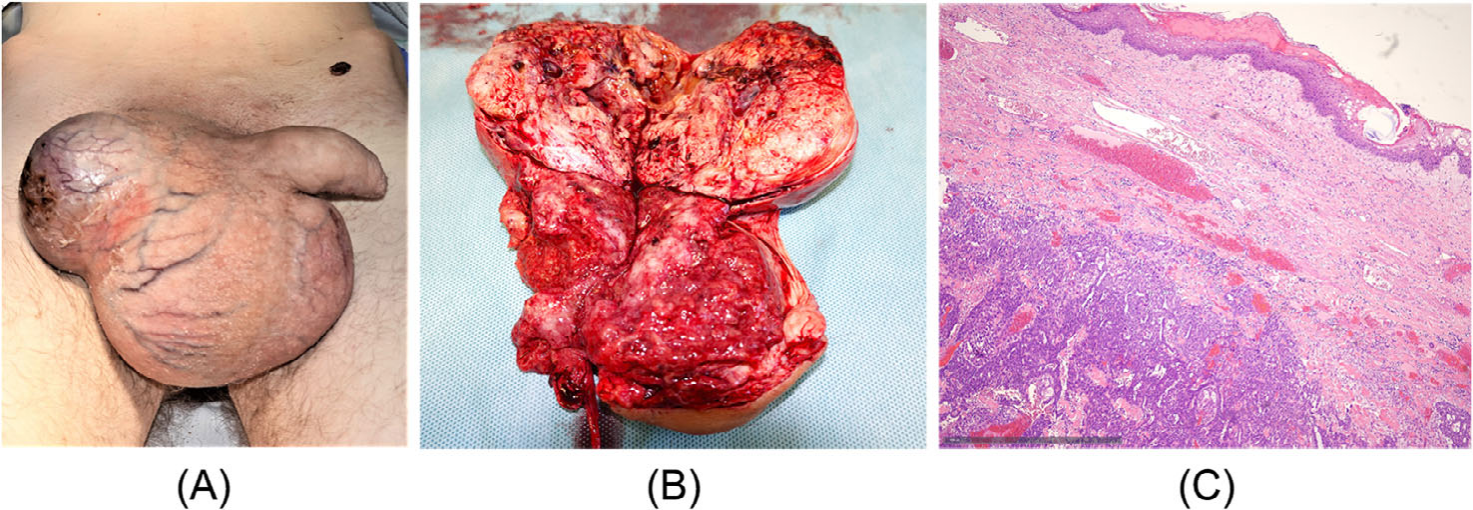
(A) Clinical appearance of right‐sided giant testicular tumour in a 29‐year‐old man (patient #3). Note the protruding tumour, close to perforating scrotal skin. (B) Gross pathologic specimen of excised testis showing inhomogenous tumour occupying the entire specimen. (C) Histologic section of the orchiectomy specimen (same patient, #3) showing polygonal and pleomorphic tumour cells with basophilic cytoplasm, solid and glandular differentiation. The cells are crowded and have indistinct cell borders as well as large nucleoli. Final histological diagnosis: mixed nonseminomatous tumour consisting of 50% teratoma, 25% yolk sac tumours and 5% choriocarcinoma. H&E staining; scale bottom left.

**FIGURE 4:**
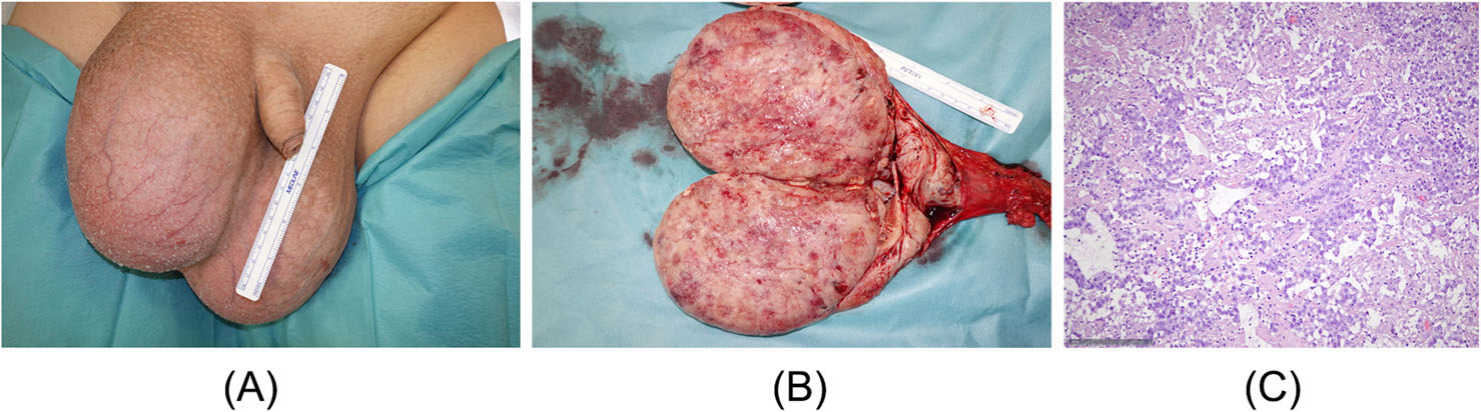
(A) Clinical appearance of left‐sided giant testicular tumour in a 31‐year‐old man (patient #4). This patient had simultaneous bilateral tumours with the left one aligning with the definition of giant tumour. The right sided tumour was 13 cm in diameter. (B) Gross pathologic specimen of excised testis showing homogeneous greyish‐white tumour mass (same patient, #4). (C) Histologic section of the orchiectomy specimen (same patient, #4) showing larger tumour cells arranged in smaller lobules as well as trabeculae with nuclei showing distinct nucleoli. Final histological diagnosis was pure seminoma. H&E staining; scale bottom left.

A total of 860 consecutive GCT patients were included, 541 with seminoma and 319 with nonseminoma. Details of the patient sample are summarized in Table 1. Four cases (0.5%; 95% confidence intervals 0.14%–1.28%) with giant testicular tumours were identified with tumour sizes ranging from 15.5 to 19 cm. Clinical details of these cases are provided in Table 2 and in Figures 1, 2, 3, 4. Histologically, two were seminomas and two nonseminomas. One patient had a second testicular tumour sized 13 cm on the contralateral side, synchronously (Figure 4). All of the four patients had metastatic disease corresponding to clinical Stage 3, three with intermediate prognosis according to IGCCCG and one with good prognosis. Two patients died, one from pulmonary embolism and one from neutropenic sepsis. All four patients admitted that they had been aware of the swelling since several months, one even reported a >1 year observation period. Reasons for delayed diagnosis remained vague. The 20‐year‐old patient (#2) reported shame. Two patients had ignored the growing mass because they did not feel any pain. The 29‐year‐old patient (#3) was a mentally disabled man working on his family's farm and he presented only when his general condition deteriorated and made him fail doing his daily farmer's work.

**TABLE 1:** Patient population: age and clinical stage distribution.

	*n* (%)
Total GCTs (*n*)	860 (100%)
Seminomas (*n*) (% of all)	541 (63%)
Nonseminomas (*n*) (% of all)	319 (37%)
Clinical stage 1 (*n*) (% of all)	639 (74%)
Clinical stage 2a and 2b (*n*) (% of all)	140 (16%)
Clinical stage 2c (*n*) (% of all)	29 (4%)
Clinical Stage 3 (*n*) (% of all)	52 (6%)
All GCT: median age, Q1–Q3 (years)	37; 31–47
Seminoma: median age, Q1–Q3 (years)	40; 33–48
Nonseminoma: median age, Q1–Q3 (years)	32; 26–38.5

*Note*: GCT germ cell tumour; Q1 first quartile; Q3 third quartile.

**TABLE 2:** Four patients with giant testicular tumours—clinical characteristics.

	#1	#2	#3	#4
Age	52 years	20 years	29 years	31 years
Histology	Seminoma	Nonseminoma (80% teratoma, 20% yolk sac tumour)	Nonseminoma (50% teratoma, 25% yolk sac tumour, 5% choriocarcinoma)	Seminoma
Largest tumour diameter	17 cm	19 cm	18 cm	15.5 cm
Laterality	Left	Right	Right	Left
Clinical stage	CS 3, intermediate prognosis	CS 3, intermediate prognosis	CS 3, intermediate prognosis	CS 3, good prognosis
Beta hCG (ref < 2.0 U/L)	210 U/L	62.4 U/L	7150 U/L	867 U/L
AFP (ref < 8.0 ng/mL)	3.1 ng/mL	5595.3 ng/mL	1376.7 ng/mL	12.7 ng/mL
LDH (ref < 250 U/L)	1490 U/L	281 U/L	922 U/L	2694 U/L
Treatment and outcome	Ox, BEPx4; CR/NED 8 years	Ox, died from pulmonary embolism 3 days postoperatively	Ox; BEPx4; DOT after fourth cycle, neutropenic sepsis	Bilateral Ox, BEPx4; CR/NED 16 months
Comment	weight loss 30 kg during last year before diagnosis	fear, shame	mentally disabled	synchronous contralateral nonseminoma (13 cm)

*Note*: Tumour marker levels represent measurements before orchiectomy.

Abbreviations: BEP, chemotherapy with bleomycin, etoposide, cisplatin; CR, complete remission; CS, clinical stage; DOT, dead of therapy; NED, no evidence of disease; ox, orchiectomy; ref, reference limit.

### 3.2 Tumour size in the present cohort of GCT patients and in subsets

The median tumour size is 32 mm in the entire GCT population while the median sizes are 30 and 35 mm in seminomas and nonseminomas, respectively (Table S1). The difference is statistically significant (*p* = 0.009). The relative frequencies of the various tumour‐size categories are provided in Table S2. The majority of GCT cases (53.4%) have tumour sizes of 2–5 cm, while only 2% of cases achieve sizes of >10 cm. Clinical stage I cases have significantly smaller tumour sizes than those with higher clinical stages (*p* < 0.001) (Table S1). In left‐sided tumours, the median size is minimally larger than in right‐sided ones (32 mm vs. 31 mm), but this difference is not statistically significant.

### 3.3 Results of the literature survey

The literature survey yielded 36 cases with giant testicular GCT ranging from 15 to 57 cm^37^ with a median size of 22 cm.^12^, ^13^, ^35^, ^36^, ^37^, ^39^, ^40^, ^41^, ^42^, ^43^, ^44^, ^45^, ^46^, ^47^, ^48^, ^49^, ^50^, ^51^, ^52^, ^53^, ^54^, ^55^, ^56^, ^57^, ^58^, ^59^, ^60^, ^61^, ^62^, ^63^, ^64^, ^65^, ^66^, ^67^ However, several reports did not specify the maximum tumour diameter but the weight of the orchiectomy specimen instead. They were included in the survey because they certainly surpassed the 15‐cm threshold. If the data of the present series are included in the list of previous cases, there appears to be a preponderance of nonseminomas among giant tumours with 24 cases opposed to 16 with seminoma. Likewise, there are more left‐sided tumours (*n* = 23) than right‐sided (*n* = 14) among the giant tumours, while one was bilateral and two were unspecified. The median age is 36 years with an interquartile range of 22–44 years. Seven cases had GCT confined to the testis (CS 1) while all others had metastatic disease, mostly CS 2c or CS 3. Twenty‐three patients of 29 with follow‐up (F/U) information were cured. However, no follow‐up (F/U) information was provided in six surviving patients, and in five cases F/U was less than 1 year. Four patients (14%) succumbed to the disease or to treatment‐related morbidities; two had questionable outcomes, while no information about follow‐up was available in 11 patients. Regarding treatment, seven patients received upfront chemotherapy for debulking followed by secondary orchiectomy. Reasons for the excessive growth of testicular neoplasms remained vague. Two patients had psychiatric disorders or were mentally handicapped; one reported shame and fear; in one patient the Covid pandemic prevented timely treatment.

## 4 DISCUSSION

The present investigation revealed a relative incidence of giant testicular germ cell tumours of 0.5% in a contemporary patient series while the median testicular tumour size is 32 mm. The literature survey revealed that the majority of giant tumours consisted of nonseminomas, histologically, and most of them presented with advanced disease (CS 2c–CS 3). Curiously, there appears to be a preponderance of the left side in giant testicular tumours.

### 4.1 Relative incidence of giant testicular tumours

Giant testicular tumours represent a rare event in the developed world where public education and health literacy rates are high. Only 0.5% (95% CIs 0.14%–1.28%) of all testicular GCTs represent giant tumours as revealed by the present investigation which is the first study to analyse the relative frequency of such cases. The frequency of 0.5% is probably a credible finding because none or only a little selection bias applies to our series, since the patient populations in the two participating institutions widely mirror the real‐world distribution of testicular cancer with no preponderance of advanced and complex cases as typically found in tertiary cancer centres. The present series derives from two Northern German testicular cancer units serving the Hamburg region and neighbouring areas by offering care for all primary testicular cancer cases. Only relapsing cases requiring high‐dose chemotherapy are transferred to tertiary oncology institutions. The median tumour size of 32 mm observed in this series is somewhat lower than recent reports from the United Kingdom, with 40 and 41 mm, respectively, and than 35 mm from Switzerland.^20^, ^22^, ^68^ Also, as documented previously, the present patient population does well compared with other representative modern series from Western countries with regard to median age, the proportion of seminomas to nonseminomas (63% vs. 37%) and the distribution of clinical stages.^18^, ^21^, ^22^, ^69^, ^70^, ^71^ In all, these data suggest that the present series does probably not involve major selection bias, and accordingly, the frequency of giant tumours of 0.5% appears to be a valid finding.

Although no clear data exist, the frequency of excessive primary tumour growths must be assumed to be higher in older series, which may be estimated from the ongoing decline of median tumour sizes over the last decades, and the concomitant decrease of diagnostic delays.^16^, ^19^, ^25^, ^72^

### 4.2 Clinical features of giant testicular tumours (literature survey)

The largest tumour size ever reported was 57 cm in a case from Wisconsin, USA.^38^ However probably, the cases presenting with weights of 5.4 and 5.27 kg, respectively, might come close to that extreme size.^50^, ^58^ The median tumour size in the group of giant tumour cases is 22 cm, which is around seven times the median size of GCT cases in our series (32 mm).^32^

The median age of 36 years among the cases with giant testicular tumours is not different from the age observed in the entire population of GCT patients in the present study (37 years), and it is consistent with the median age reported in large recent patient series.^22^, ^73^ However, the frequency of nonseminomas of 60% among giant testicular tumours is higher than the average incidence of 36%–40% observed in contemporary GCT series.^70^, ^71^, ^73^ A history of undescended testis was reported in two of the cases with giant GCTs.^52^, ^57^ Thus, the frequency of that feature in giant testicular tumours would be 5%, which is not distinctly different from the rate of 8%–10% found in the general population of testicular GCTs. A summary of literature review on giant testicular germ cell tumours is in Tables 3 and 4.

**TABLE 3:** Literature survey: giant testicular germ cell tumours reported since 1950.

First author	Reference	Year	Country	Age	Side	Tumour size (cm)	Histology	Clinical stage	Outcome (F/U time‐span)	Comment
Betts	40	1954	UK	40	Left	18	NS	CS 3	DOD (4 weeks)	
Pollack	41	1962	USA	61	R	25	SE	CS 1	CR (3 years)	No adjuvant therapy
Kademian	38	1976	USA	59	R	57 (2730 g)	NS	CS 1 (RPLND)	CR (6 years)	>6 months history; concomitant hernia disguising tumour
Thackray	21	1964	UK	n.a.	n.a	30	SE	n.a.	n.a.	
Pugh	13	1964	UK	n.a.	n.a	16	NS	n.a.	n.a.	
Pannek	42	1996	Germany	25	Left	Man's head size	SE	CS 2c	Multiple relapses, CR (18 months)	6 months diagnostic delay; upfront chemotherapy
Saiko	43	1992	Japan	66	Left	26	SE	CS 2b	n.a.	Neglected growing tumour for 5 years
Hyouchi	44	1997	Japan	33	Left	15	NS	CS 3, poor pr.	CR (4 months)	
Kin	45	1999	Japan	38	Left	32	NS	CS 3,	CR (13 months)	Upfront chemotherapy
Lo	46	2001	Canada	n.a.	Left	(1.6 kg)	SE	CS 2c	CR (24 months)	Patient neglected growth for 5 years; scrotal orchiectomy
Fujita	67	2001	Japan	19	Left	(1.7 kg)	NS	CS 3	CR (21 months)	
Tomaskovic	47	2004	Croatia	21	Left	29	NS	CS 3, poor pr.	CR (F/U n.a.)	
Nabi	48	2002	India	18	R	>15	NS	CS 2c	CR (F/U n.a.)	Upfront chemotherapy
Al Assiri	49	2005	Canada	35	Left	14.5 cm	NS	CS 1	CR (14 months)	Two years diagnostic delay
Zangana	50	2007	Iraq	36	Left	(1.926 kg)	NS	CS 3	CR (32 months)	
Letsch	51	2010	Germany	36	bilateral	>15	SE	CS 2c	CR (8 months)	Upfront chemotherapy
Grigore	52	2012	Romania	42	Left	15	SE	n.a.	n.a.	>12 months growing mass neglected
Ellimoottil	53	2012	USA	42	Left	31	SE	CS 1	CR (1 year)	surveillance after orchiectomy
Alaskari	54	2013	Saudi Arabia	53	Left	22	NS	CS 3	n.a.	
Szturz	55	2013	Czech Republic	53	R	30	SE	CS 3	CR (5 months)	Upfront chemotherapy
Ozaki	56	2013	Japan	36	R	(1.926 kg)	SE	CS 1	CR (19 months)	
Khosla	57	2014	India	16	R	22	NS	CS3	n.a.	Upfront chemotherapy
Nomura	58	2014	Japan	55	R	(5.27 kg)	SE	CS 2c	n.a.	
Nakaya	59	2016	Japan	25	Left	18	SE	CS 2c	n.a.	
Reekhaye	60	2016	UK	23	Left	(2.47 kg)	NS	CS 3	CR (4 months)	Fear, shame and embarrassment
Chira	61	2017	Romania	44	R	40	NS	CS 3	n.a.	
Iguchi	62	2018	Japan	51	Left	(3.4 kg)	NS	CS 2c	CR (3 years)	
Jackson	39	2020	Australia	22	Left	25	NS	CS 3	PR (F/U n.a.)	
Majewska	63	2022	Poland	30	R	21.5	NS	CS 3, poor pr.	n.a.	
Stock	64	2021	Germany	47	Left	22	NS	CS 2c, poor pr.	CR (F/U n.a.)	Upfront chemotherapy
Antonaci	65	2021	Italy	44	Left	28	NS	CS 2c	Relapse (18 months)	
De Luca	35	2023	Italy	36	Left	17	SE	CS 1	CR (F/U n.a.)	Psychiatric disorder
Aizat Sabri	36	2023	Malaysia	21	R	30 (2.14 kg)	NS	CS 2c	n.a.	Diagnostic delay through Covid pandemy
Tariqi	37	2024	Morocco	75	R	32	NS	CS 3, poor pr.	CR (12 months)	Orchiectomy with excision of scrotal skin
Omorphos	66	2025	UK	43	Left	35	SE	CS 1	CR (8 months)	7 years diagnostic delay; scrotal orchiectomy
Present report		2025	Germany	52	Left	17 (1.67 kg)	SE	CS 3, intermediate pr.	CR (8 years)	1 year diagnostic delay
		2025	Germany	20	R	19 (2.41 kg)	NS	CS 3, intermediate pr.	Died from pulmonary embolism (3 days after surgery)	
		2025	Germany	29	R	15	NS	CS 3, intermediate pr.	Died from neutropenic sepsis (4 th cycle of chemotherapy)	Mentally disabled
		2025	Germany	31	Left	15.5	SE	CS 3, good pr.	CR (16 months)	in addition: right side 13 cm seminoma

Abbreviations: CR, complete remission; CS, clinical stage; DOD, dead of disease; F/U, follow up; n.a., not available; NS, nonseminoma; pr, prognosis; R, right side; RPLND, retroperitoneal lymph node dissection; SE, seminoma.

**TABLE 4:** Summary of literature review on giant testicular germ cell tumours.

Total number of giant testicular GCT	40
Median age (years)	36, IQR 22–44, range 19–75
Laterality (*n*)	23 left/14 right sided
Histology (*n*)	24 NS/16 SE
Presenting clinical stage (*n*)	7 CS1; 30 > CS1, 3 n.a.
Treatment (*n*)	34 orchiectomy 6 upfront chemotherapy
Outcome (*n*)	23 cured (80%) 4 died (14%) 13 incomplete or n.a.

Abbreviations: GCT, germ cell tumour; IQR, interquartile range; n.a., not available; NS, nonseminoma; SE, seminoma.

Amazingly, seven patients presented with clinical stage 1 disease despite their huge primary tumours. Five of the CS1 cases had seminoma, histologically, and this finding is in contrast to the current understanding that primary tumour size in seminoma is closely associated with the frequency of metastatic spread.^74^ There is no clear explanation why these giant seminomas remained confined to the testis, but the finding is in line with the word of wisdom that exceptions may occur from any rule in clinical medicine.

All other patients with giant testicular tumours where clinical staging was available, had metastatic disease. This finding is consistent with the observation of significantly larger tumour sizes in cases with CS > 1 revealed in this series and it aligns with the general experience that larger GCTs are more frequently metastasized than small primary tumours.^15^, ^32^, ^69^

### 4.3 Laterality of giant testicular tumours

A curious finding is the marked preponderance of left‐sided tumours (23 left vs. 14 right; L:R = 1.64) among cases with giant testicular tumours. This observation would align with a previous report that noted a significantly larger median size of left‐sided GCTs opposed to right‐sided ones.^21^ In the present GCT series, the median tumour size of left‐sided tumours (32 mm) is also marginally larger than that of right‐sided GCTs (31 mm), but this difference is not significant, statistically. Generally, laterality appears to be a relevant factor in testicular biology, since, anatomically, the venous drainage of the testes is different between the two sides. Further, the right testis is on average slightly larger than the left one in healthy males, and undescended testes are more common on the right side than on the left.^75^, ^76^ Also, in most of the larger GCT patient series, there is a near 5:4 ratio in favour of right‐sided tumours, which is also found in the present series (51% vs. 49%).^14^, ^77^, ^78^, ^79^ There is no biological explanation at hand for the putative predisposition of giant testicular tumours to the left side. A chance finding appears likely, particularly because the overall number of giant GCTs is still rather small. Yet, the observation appears noteworthy because it might generate hypotheses regarding the relevance of laterality in testicular GCTs if confirmed in further studies.

### 4.4 Treatment of giant testicular tumours

Radical orchiectomy was the first step of treatment in 33 cases with giant tumours (83%), and this was also done in the four cases of the present series. Usually, extended exposure with lengthening the inguinal incision down to the scrotum is required to remove extraordinarily large testicular masses. In most cases, additional excision of scrotal skin is needed to tailor redundant scrotal skin, or to excise GCT protruding to the skin (illustrated in Figure 2B).^37^, ^60^ In isolated cases, scrotal orchiectomy was performed for technical reasons.^66^ By contrast, seven of the reported cases with giant GCT underwent upfront chemotherapy for debulking tumour mass before secondary surgery. This is probably a rational approach, particularly, in cases where concomitant visceral metastases require urgent treatment. On the other hand, giant testicular tumours are well suited to surgery because they are located outside of the abdominal cavity and can usually be removed by a rather simple surgical procedure. The advantage of primary orchiectomy would be the immediate and considerable reduction of the overall tumour burden. The delay to the start of chemotherapy imparted by surgery is usually no longer than a couple of days and would thus not compromise the overall outcome. But clearly, in cases where the tumour has invaded neighbouring structures and when local tumour control cannot safely be achieved by surgery, upfront chemotherapy would be the treatment of choice.

### 4.5 Therapeutic outcome in giant testicular tumours

Almost 80% of patients with giant GCT were cured. However, this high cure rate is based on only the 29 cases with full information about survival. Noteworthy, no data about F/U time was available in six cases, and in five other cases, the F/U time was less than 1 year. The markedly high cure rate is surprising because most of the patients had advanced disease stages with some of whom belonging to the IGCCCG poor prognosis group. Moreover, many of the cases obviously featured a high‐risk profile for relapsing disease. Therefore, the 80% cure rate must probably be considered with caution particularly, because of the short F/U time in a substantial number of patients.

Reporting bias is likely one explanation for the favourable outcome in the majority of cases, since most of the reports came from experienced cancer centres where the chance of cure is significantly superior to that in smaller institutions.^24^, ^80^ Also, the chance of reporting is much higher in academic centres than in small regional or local institutions, particularly, when cases were managed successfully. It is of note that fatalities may not only occur from the progression of cancer but also from complications of treatment like neutropenic sepsis or cancer‐associated thrombosis with pulmonary embolism as observed in two patients of the present series.

### 4.6 Reasons for extensive tumour growth

Diagnostic delay is the most likely reason for the occurrence of giant testicular tumours. All of the four cases of our own institutions reported that they had noticed the scrotal swelling many months before they sought medical advice. Three cases included in the literature survey had noticed the new growth even more than 5 years before seeing a doctor while several others had symptomatic intervals of more than 6 months.^43^, ^46^, ^66^ However, the underlying reasons why the patients tolerated the growing scrotal mass remain obscure. Negligence in the absence of pain could be one possible reason for not immediately seeking medical assistance.

One of our own patients reported shame preventing him from seeking professional help and the same is reported from one patient from the United Kingdom.^60^ Two of the patients included in the survey were mentally disabled, and they probably did not realize the health risk originating from the growth.

The majority of cases with giant tumours did not report clear reasons for delaying the diagnosis. Therefore, one can only assume a triad of unawareness of testicular maladies, negligence and possibly shame keeping patients away from medical help while the testicular mass is constantly growing. In all, it is an enigma why giant testicular tumours do still occur even in developed countries with health care systems offering service even to economically less privileged individuals.^81^ To avoid such events, information campaigns already launched need to be continued and preferably intensified.^33^, ^82^, ^83^

### 4.7 Limitations of the study

As any retrospective study, this investigation cannot exclude some selection bias, because some cases might have been missed in the analysis. The literature survey might be incomplete because we only searched for articles with abstracts in the English language. Therefore, reports published in local journals not listed in international data bases were not identified with the present search strategy.

## 5 CONCLUSIONS

Although the median size of testicular germ cell tumours is decreasing for decades, giant testicular tumours (>15 cm in diameter) still occur with a relative frequency of 0.5% of all GCTs. Most of the cases with such giant tumours have advanced disease. Primary orchiectomy is usually the first step of treatment, but upfront cisplatin‐based chemotherapy may be required if local tumour control cannot be achieved by surgery. Most of the cases can be cured by a combination of surgery and chemotherapy. Curiously, there appears to be a preponderance of left‐sided cases in giant testicular tumours. Delayed diagnosis is the likely reason that lets the neoplasms grow excessively, but the underlying reasons keeping patients away from professional help remain elusive. Information campaigns employing modern communication technologies and including individuals from socioeconomically underprivileged groups should be supported to increase men's awareness of genital diseases.^33^, ^34^, ^84^, ^85^

## AUTHOR CONTRIBUTIONS

*Conceptualisation*: Markus Angerer and Klaus‐Peter Dieckmann. *Original draft*: Markus Angerer and Klaus‐Peter Dieckmann. *Data curation and analysis*: Alexander C. Harms, Markus Angerer, Klaus‐Peter Dieckmann. *Supervision*: Christian Wülfing. *Review and editing*: All authors.

## CONFLICT OF INTEREST STATEMENT

The authors have no relevant financial or nonfinancial interests to disclose.

## Supporting information


**Table S1.** Median tumour sizes in entire population and in subgroups.

**Table S2:** Relative frequencies of tumour‐size categories.

## Data Availability

The data that support the findings of this study are available from the corresponding author upon reasonable request.
