# Supplementary material for: Giant testicular germ‐cell tumours—An analysis of relative incidence and clinical features based on a clinical case series and a survey of the literature
Source: BJUI Compass. 2026 Jan 12;7(1):e70118. doi: 10.1002/bco2.70118 (PMC12795782; doi:10.1002/bco2.70118)
Supplement: Supplementary file 1 — Table S1. Median tumour sizes in entire population and in subgroups. [file BCO2-7-e70118-s001.docx]

**Supplementary Table 1**

**Median tumour sizes in entire population and in subgroups**

|  | n**(%)** | **Median size (mm)** | **Q1 (mm)** | **Q3 (mm)** | **P value** |
| --- | --- | --- | --- | --- | --- |
| **GCT (all)** | 860 (100%) | 32 | 20 | 47 | - |
| **Seminoma** | 541 (63.6%) | 30 | 19 | 46 | - |
| **Nonseminoma** | 319 (37%) | 35 | 22 | 49 | **0.009**^1^ |
| **Clinical stage 1** | 639 (74%) | 32 | 20 | 47 | - |
| **Clinical stage >1** | 221 (26%) | 38 | 25 | 53.5 | **<0.001^1^** |
| **Left-sided GCTs** | 426 (49%) | 32 | 20 | 47 | ^-^ |
| **Right-sided GCTs** | 434 (51%) | 31 | 19 | 48 | 0.8 ^1^ |

^1^Mann-Whitney U test;
