# Supplementary material for: Giant testicular germ‐cell tumours—An analysis of relative incidence and clinical features based on a clinical case series and a survey of the literature
Source: BJUI Compass. 2026 Jan 12;7(1):e70118. doi: 10.1002/bco2.70118 (PMC12795782; doi:10.1002/bco2.70118)
Supplement: Supplementary file 2 — Table S2: Relative frequencies of tumour‐size categories. [file BCO2-7-e70118-s002.docx]

**Supplementary Table 2:**

**Relative frequencies of tumour-size categories**

| Tumour-size | **<20 mm** | **20–50 mm** | **51–100 mm** | **101–150 mm** | **>150 mm** |
| --- | --- | --- | --- | --- | --- |
| **All GCT (n=860)** | 207 (24.1%) | 459 (53.4%) | 177 (20.6%) | 13 (1.5%) | 4 (0.5%) |
| **Seminoma (n=541)** | 139 (25.7%) | 287 (53.0%) | 106 (19.6%) | 7 (1.3%) | 2 (0.4%) |
| **Nonseminoma (n=319)** | 68 (21.3%) | 172 (53.9%) | 71 (22.3%) | 6 (1.9%) | 2 (0.6%) |
